# Transactional sex among young women in rural South Africa: prevalence, mediators and association with HIV infection

**DOI:** 10.7448/IAS.19.1.20749

**Published:** 2016-07-27

**Authors:** Meghna Ranganathan, Lori Heise, Audrey Pettifor, Richard J Silverwood, Amanda Selin, Catherine MacPhail, Sinead Delany-Moretlwe, Kathleen Kahn, F Xavier Gómez-Olivé, James P Hughes, Estelle Piwowar-Manning, Oliver Laeyendecker, Charlotte Watts

**Affiliations:** 1 Department of Global Health and Development, Faculty of Public Health and Policy, London School of Hygiene and Tropical Medicine (LSHTM), London, UK; 2 Wits Reproductive Health and HIV Institute, University of Witwatersrand, Johannesburg, South Africa; 3 Department of Epidemiology, University of North Carolina at Chapel Hill, Chapel Hill, NC, USA; 4 Carolina Population Center, University of North Carolina, Chapel Hill, NC, USA; 5 MRC/Wits Rural Public Health and Health Transitions Unit (Agincourt), Faculty of Health Sciences, School of Public Health, University of the Witwatersrand, Johannesburg, South Africa; 6 Department of Medical Statistics, Faculty of Epidemiology and Population Health, London School of Hygiene and Tropical Medicine (LSHTM), London, UK; 7 School of Health, University of New England, Armidale, Australia; 8 Division of Epidemiology and Global Health, Department of Public Health and Clinical Medicine, Umeå Centre for Global Health Research, Umeå University, Umeå, Sweden; 9 International Network for the Demographic Evaluation of Populations and Their Health (INDEPTH) Network, Accra, Ghana; 10 Department of Biostatistics, University of Washington, Seattle, WA, USA; 11 Department of Pathology, HPTN Laboratory Center, Johns Hopkins University School of Medicine, Baltimore, MD, USA; 12 Laboratory of Immuno-Regulation, NIAID, NIH, Baltimore, MD, USA; 13 Department of Medicine and Epidemiology, Johns Hopkins University, Baltimore, MD, USA

**Keywords:** transactional sex, structural drivers, HIV, adolescent women, young women, sub-Saharan Africa, risky sexual behaviours

## Abstract

**Introduction:**

Young adolescent women in sub-Saharan Africa are three to four times more likely to be HIV-positive than boys or men. One of the relationship dynamics that is likely to be associated with young women's increased vulnerability to HIV is transactional sex. There are a range of HIV-related risk behaviours that may drive this vulnerability. However, to date, limited epidemiological data exist on the role of transactional sex in increasing HIV acquisition, especially among young women in sub-Saharan Africa. Our paper presents data on the prevalence of self-reported engagement in transactional sex and explores whether transactional sex is associated with increased risk of HIV infection among a cohort of young, rural, sexually active South African women. We also explore whether this relationship is mediated through certain HIV-related risk behaviours.

**Methods:**

We analyzed baseline data from a phase III trial of conditional cash transfers for HIV prevention of 693 sexually active, school-going young women aged 13–20 years in rural South Africa. We examined the association between young women's engagement in transactional sex and HIV infection. Transactional sex is defined as a non-commercial, non-marital sexual relationship whereby sex is exchanged for money and/or gifts. We explored whether this relationship is mediated by certain HIV-related risk behaviours. We used logistic and multinomial regression and report unadjusted and adjusted odds ratios with 95% CI.

**Results:**

Overall, 14% (*n*=97) of sexually active young women reported engaging in transactional sex. Engagement in transactional sex was associated with an increased risk of being HIV-positive (aOR: 2.5, CI: 95% 1.19–5.25, *p*=0.01). The effect size of this association remained nearly unchanged when adjusted for certain other dimensions of HIV risk that might help explain the underlying pathways for this relationship.

**Conclusions:**

This study provides quantitative support demonstrating that transactional sex is associated with HIV infection in young women. Even though the specific variables tested do not mediate the relationship, a potential explanation for this association may be that the men with whom young women are having sex belong to networks of sexually connected individuals who are at a “high risk” for HIV infection. The results highlight the importance of structural intervention approaches that can alter the context of young women's HIV risk.

## Introduction

The HIV epidemic in South Africa is one of the largest in the world [1,2] and is largely heterosexually transmitted [3]. Young women of child-bearing age have a significantly higher HIV prevalence (5.6% vs. 0.7%) [4–6] and incidence (2.5% vs. 0.6%) [4,7,8] than males of the same age. In addition to increased biological vulnerability of young women, relational risk factors, such as age-disparate relationships, engagement in transactional sex and violence within partnerships [9–11], as well as individual risk behaviours – such as inconsistent condom use, number of partners and age at sexual debut – have been found to be associated with young women's risk of HIV infection [12].

Transactional sex has received increasing attention in the public health literature, as it is believed to be an important contributing factor to the high HIV infection rates observed among young women in sub-Saharan Africa [13,14]. There is currently a wide-ranging debate on the definition of transactional sex, but it is defined here as a non-marital sexual relationship where men and women exchange sex for, or in anticipation of, material possessions or favours (such as money, clothing, transportation and school fees). It is considered to be sex framed outside of prostitution or sex work by those who participate in the exchange and can be differentiated by the negotiating process, that is, in transactional sex there is no up-front negotiation or pre-determined payment and a wide range of goods (money, but also gifts, favours) may be exchanged [13], whereas in sex work, there is an explicit up-front negotiation of the terms of the exchange [10]. In addition, women engaging in transactional sex seldom identify themselves as sex workers. Reflecting economic and social roles within many high HIV prevalence countries, it is predominantly men who provide and women who receive these material benefits in transactional sexual encounters [10,15–17]. This dynamic might in turn render young women vulnerable to HIV. Reporting of transactional sex is varied as indicated by evidence from population-based Demographic and Health Surveys (DHS) data of transactional sex in the past year from 12 sub-Saharan African countries which suggest that the prevalence of transactional sex ranges from 2 to 26.6% across settings [18]. The academic literature highlights that factors associated with transactional sex are complex; demographic and socio-economic factors can be an important determinant, with young women using sex to access essential resources, including food and school fees. In addition, peer or family pressure, as well as young women's aspirations for acquiring expensive goods or connections to boost their status may also be important motivating factors [19,20].

It is not just the transactional aspect that makes such sexual encounters potentially risky for HIV acquisition. Transactional sex might overlap with a range of factors that have been shown to be associated with HIV acquisition – such as sexual relations between a younger woman and an older man (who is more likely to be HIV-positive) [9,21,22], sex under the influence of alcohol or drugs [23,24], having multiple sexual partners or engaging in a relationship with a man who concurrently has other partners [25–27]. Together, these factors might reflect aspects of a transactional relationship that may make young women vulnerable to HIV infection. In addition, partnership dynamics, such as unequal power within a relationship, may undermine condom use thereby increasing HIV risk [28–30].

Despite the potential for transactional sex to increase HIV risk, there is limited quantitative data demonstrating an association in young women: only two studies, both from South Africa, showed evidence of an association in young women [10,31]. Results from the cross-sectional analysis of a quasi-experimental community-based survey in Kwa-Zulu Natal and Eastern Cape provinces involving 2624 young women, aged 15–24 years, showed that young women who reported having engaged in transactional sex have almost twice the odds of being HIV seropositive as compared with those who do not report engaging in transactional sex [31]. In another prospective cohort study of South African women (*n*=1077) aged 15–26 years, Jewkes *et al*. found that young women who reported having transactional sex with a once-off partner or with an ongoing secondary partner had higher HIV incidence than those not engaging in transactional sex (this result remained after adjusting for number of partners and age difference between partners) [10].

To help address this shortage of quantitative studies examining the relationship between transactional sex and HIV infection, particularly in young women, our paper presents data on the prevalence of self-reported engagement in transactional sex and examines whether transactional sex is associated with an increased risk of HIV infection among a cohort of young, rural, sexually active South African women. We also examine whether this relationship is mediated through certain HIV-related risk behaviours.

## Methods

### Study setting and data collection

This paper is a secondary analysis of cross-sectional data collected during baseline interviews with participants from a phase III, individually randomized conditional cash transfer (CCT) trial in rural South Africa (HPTN 068) [32,33]. Participants at the baseline interview were sexually active, school-going young women who reported ever having had vaginal and/or anal sex. Data collection was conducted from March 2011 to December 2012 in the sub-district of Agincourt in rural Mpumalanga Province, northeast South Africa, an area with high levels of poverty, unemployment and labour migration [34–36]. The Medical Research Council (MRC)/Wits University Rural Public Health and Health Transitions Research Unit runs the Agincourt Health and Socio-Demographic Surveillance System (AHDSS) in this area, and this was the platform for identifying eligible households and young women [37]. The purpose of the trial was to determine whether providing cash transfers to young women and their households – conditional on school attendance – reduces HIV incidence among young women. The intervention involved individually randomizing young women aged 13–20 years to receive a monthly cash transfer, conditional on school attendance. Study participants were eligible for inclusion in the trial if they were females aged 13–20 years; enrolled in grades 8, 9, 10 or 11 at selected schools in the AHDSS study site; and had a bank or post office account to receive the transfer. The participants were excluded if they were pregnant or married at baseline. Both parental/legal guardian consent and young woman consent/assent were required to participate. As part of the enrolment process, after completing the baseline interview, young women underwent pre-test counselling and then blood samples were collected and tested for HIV and HSV-2 infection. The total sample size of the trial was 2533 young women and their parent/guardian (with one young woman per household enrolled); the sample subset for this paper was 693 sexually active young women.

The exposure variable is young women who report engaging in transactional sex and the outcome variable is HIV infection.

Ethical approval for the secondary analysis was provided by the London School of Hygiene and Tropical Medicine Research Ethics Committee, and for the main trial by the University of North Carolina at Chapel Hill Institutional Review Board, the Human Research Ethics Committee (Medical) at the University of Witwatersrand, Johannesburg, and the Departments of Health and Education, Mpumalanga Province, South Africa, where the research was conducted.

### Measurement tools

Young women completed computer-based questionnaires which were primarily self-administered using Audio-Computer Assisted Self-Interviewing (ACASI) and parents/guardians completed interviewer-administered, structured, computer-based household questionnaires. Information on household and socio-economic characteristics (household questionnaire) and socio-demographic background, sexual experiences and partner history (young women's questionnaire) were included in the questionnaires. Due to the personal nature of some of the questions in the young women's questionnaire (i.e. details of sexual relationships), these questions were filled out by young women directly. Both the parent/guardian and young woman's interviews were conducted in the language preferred by the participant – in the local language, xiTsonga, or English. The questionnaires were translated into xiTsonga by bilingual researchers and checked for linguistic appropriateness, comprehension and cultural relevance and then back-translated from xiTsonga into English to ensure accuracy and fidelity to meaning.

### Conceptual framework and variables

**Figure 1:**
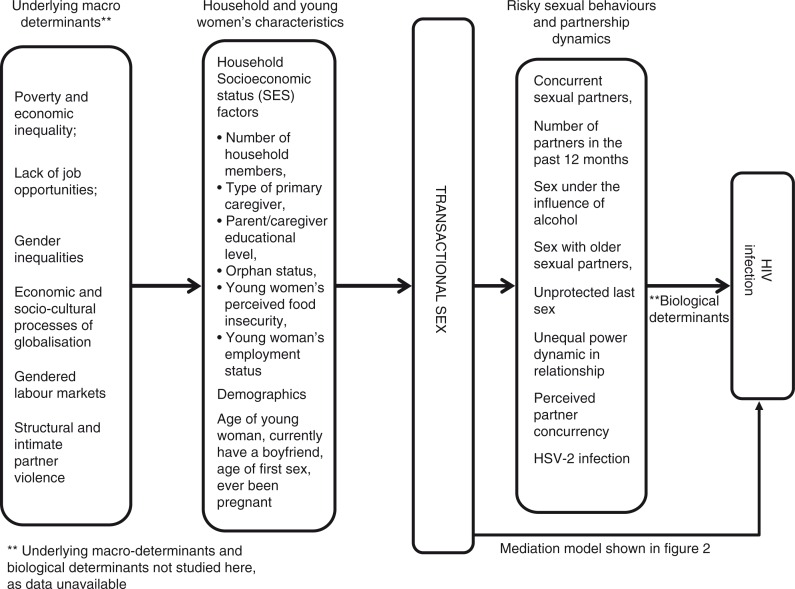
Underlying and proximate determinants associated with transactional sex and pathways through which transactional sex affects HIV risk.

We use a modified version of the proximate-determinants framework [38] (see Figure 1) which acknowledges underlying structural and proximate factors that contribute to HIV risk, to guide our selection of confounding and mediating variables. Our conceptual framework recognizes the influence of factors such as demographic and socio-economic factors on young women's engagement in transactional sex and how certain partner dynamics or relationship characteristics might potentially mediate the relationship between transactional sex and HIV infection. While this paper is focused on the relationship between young women's engagement in transactional sex and HIV infection, a forthcoming paper explores socio-demographic factors associated with young women's engagement in transactional sex and was part of the first author's doctoral research [39].

#### Biological variables

The outcome variable *HIV serostatus* at baseline was assessed with two HIV rapid tests [40] done in parallel with the FDA-approved Uni-gold™ Recombigen^®^ HIV (Trinity Biotech plc, Bray, Co. Wicklow, Ireland) and Determine™ HIV-1/2 (Alere Medical Co.Ltd, Matsudo-shi, Chiba, Japan*)* test. If both of the HIV rapid tests were non-reactive, no further testing was done. If one or both of the HIV rapid tests was reactive, a CD4 cell count was performed and confirmatory test was performed using an FDA-cleared Western blot test. Further details on HIV testing have been described in the HPTN 068 study protocol and the baseline paper [32,33]. *HSV-2 infection* testing was performed using the Herpes Simplex Virus Type 2 IgG ELISA assay (Kalon Biological, LTD Guildford, UK), with an index cut-off of 1.5 normalized optical density units. If the HSV-2 test was positive, no further HSV-2 testing was done at the study site at follow-up visits. HSV-2 results were confirmed retrospectively at the HPTN Laboratory Centre.

#### Main exposure variable

The main exposure variable was “having had transactional sex,” shortened to “transactional sex” and coded as a binary variable (yes/no) for sex in exchange for money and/or gifts. We asked the young woman about her sexual and relationship history with her three most recent partners, starting with the most recent partner. The four steps carried out to derive the transactional sex variable were:

1. Variable “transactional sex for money” coded 1 if participant said yes to “Did you feel like you had to have sex with [initials] because they gave you money”?;
2. Variable “transactional sex for gifts” coded 1 if participant said yes to “Did you feel like you had to have sex with [initials] because they gave you things (such as airtime, cell phone, groceries, clothes or shoes, perfume or lotions, make-up, cool-drinks, sweets or chips, CDs, DVDs or videos, alcohol or drugs, flowers, other (specify))”?;
3. Variable “transactional sex for money and gifts” coded 1 if participant had said yes to question (1) “Did you feel like you had to have sex with [initials] because they gave you money”? *and* question (2) “Did you feel like you had to have sex with [initials] because they gave you things”?;
4. The final variable “transactional sex for money and/or gifts” coded 1 if participant said yes to “Did you feel like you had to have sex with [initials] because they gave you money”? *or* “Did you feel like you had to have sex with [initials] because they gave you gifts *or* both gifts and money”?

#### Mediating variables

**Figure 2:**
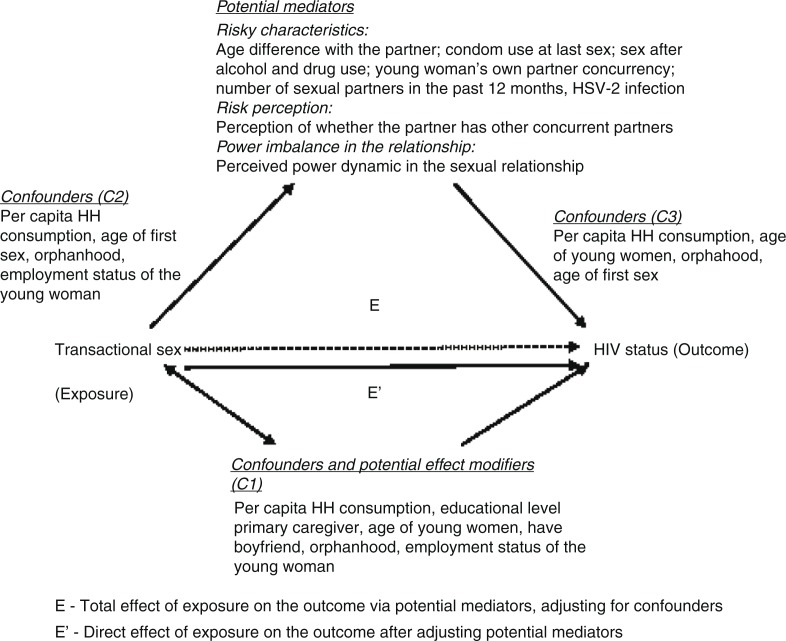
Hypothesized mediation model between young women's engagement in transactional sex and HIV infection.

Potential mediators around partner characteristics and certain relationship dynamics were selected based on a review of the literature, as shown in our proximate-determinants conceptual framework (Figure 1) and their possible role as mechanisms through which transactional sex works to affect HIV infection. These are age difference between partners, condom use at the last sexual encounter, sex under the influence of alcohol or drugs, partner concurrency by young women and her perception of partner concurrency, number of sexual partners in the past 12 months and sexual relationship power dynamics [9,23,41]. In addition, we included HSV-2 infection in the mediation analysis as young women engaging in transactional sex are more likely to be HSV-2 infected [42] and HSV-2 infection has shown to increase the risk of HIV infection [43]. Figure 2 illustrates the hypothesized mediation conceptual model between young women's engagement in transactional sex and HIV. We have described the construction of each of these variables in detail in Appendix 1.

#### Other variables

We selected the following variables from the conceptual framework as confounders based on the literature and our bivariate analysis: the age of young women, age of first sex, employment status of young women, per capita household consumption (as a measure of household living standards), educational level of primary caregiver and orphan status. Please see Appendix 1 for details on how each variable was constructed.

### Missing data

**Figure 3:**
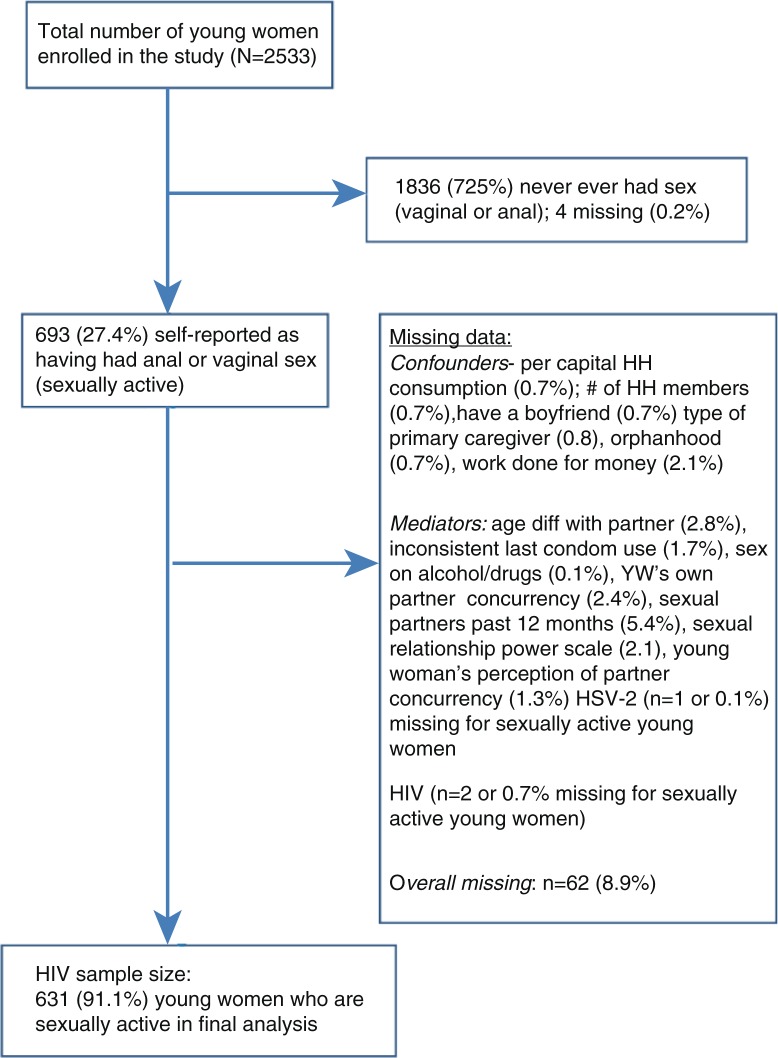
Flowchart for final sample size in analysis between transactional sex and HIV infection.

There were little missing data in this dataset. With the exception of the variable, *number of sexual partners in the past 12 months* (where missing data were ~5%), almost all the exposure variables had less than 3% missing data. This includes cases where young women have “refused to answer.” The response “don't know” was also coded as missing, as the percentage of this response code was exceedingly small. No attempt was made to replace missing data and only individuals with complete data were included in the final models. Please see Figure 3 for flowchart on final sample size.

### Statistical analysis

Descriptive statistics were used to summarize the socio-demographic characteristics of the sample (see Table 1) and the prevalence and patterns of young women ever having engaged in transactional sex. Logistic regression models were fitted to obtain unadjusted odds ratios for the relationship between self-reported transactional sex and HIV infection.

**Table 1:** Selected socio-demographic, partnership characteristics and sexual behaviours among sexually active young women (aged 13–20 years) (*n*=693)

	Sexually active (%)
**Socio-demographic characteristics**	
Age of young woman (*n*=693)	
13–15 years	151 (21.8)
16–20 years	542 (78.2)
Per capita household consumption^a^ (*n*=693)	
Low	220 (31.7)
Medium	279 (40.3)
High	194 (28.0)
Number of household members (*n*=693)	
2–3 members	87 (12.5)
4–5 members	233 (33.6)
6–7 members	220 (31.7)
≥ 8 members	153 (22.1)
Type of primary caregiver (*n*=692)	
Mother	471 (68.1)
Father	22 (3.2)
Brother/sister	65 (9.4)
Other blood relative	134 (19.4)
Educational level of primary caregiver (*n*=692)	
None	176 (25.4)
Primary	196 (28.3)
Secondary	164 (23.7)
Matric or tertiary	128 (18.5)
Adult basic education	28 (4.1)
Orphan status (*n*=684)	
Parents alive	475 (69.4)
One or both parents dead	209 (30.6)
Young women's perceived food insecurity^b^ (*n*=684)	
No	412 (60.2)
Yes	272 (39.8)
**Partnership characteristics and sexual behaviours**	
Currently have a boyfriend (*n*=693)	
No	151 (21.8)
Yes	542 (78.2)
Lifetime sexual partners (*n*=648)	
1	353 (54.5)
2	163 (25.1)
3	61 (9.4)
4–11	71 (11.0)
Sexual partners in the past 12 months (*n*=660)	
1	520 (78.8)
2	97 (14.7)
> 3	43 (6.5)
Age of first sex (*n*=634)	
Up to 15 years	127 (20.0)
15 years and above	507 (80.0)
Ever been pregnant (*n*=663)	
No	460 (69.4)
Yes	203 (30.6)
**Transactional sex**	
Transactional sex (*n*=693)	
No	596 (86)
Yes	97 (14)
Breakdown of percentages by money	
or gifts or both^c^ (*n*=97)	
Sex in exchange for money	58 (59.8)
Sex in exchange for gifts	24 (24.7)
Sex in exchange for money and gifts	15 (15.5)
**Employment characteristics**	
Work done for money (*n*=683)	
No	534 (78.2)
Yes	149 (21.8)
Main reasons for working (*n*=147)	
Want money for myself	82 (55.8)
Support my family	37 (25.2)
Something to do	25 (17.0)
Way to meet friends	3 (2.04)
Primary type of work (*n*=147)	
Sewing, hair, baking and brewing	29 (19.7)
Child care	18 (12.2)
Factory worker	14 (9.5)
Working in a shop	12 (8.2)
Other	12 (8.2)
Small business assistant	11 (7.5)
Domestic worker	11 (7.5)
Mining	9 (6.1)
Clerical and office work	8 (5.4)
Transport	6 (4.1)
Farm worker	5 (3.4)
Informal selling	5 (3.4)
Sex work	4 (2.7)
Tavern or restaurant	2 (1.4)
Tourism/game parks	1 (0.7)
Primary source of money in the past 12 months	
Family	203 (30.2)
Job	180 (26.8)
Didn't have any money	73 (10.9)
Grants (child support, disability)	69 (10.3)
Boyfriend or partner	51 (7.6)
Friends	35 (5.2)
Begging/shoplifting, etc.	21 (3.1)
Sex work	18 (2.7)
Other	12 (1.8)
Selling drugs	9 (1.3)

a Measure of household living standards

b young women worried about having enough food for her and her family in the past 12 months

c among sexually active young women who responded yes to question on transactional sex.

Using logistic or multinomial logistic regression for binary and categorical mediators, respectively, we calculated odds ratios for the relationship between transactional sex and each potential mediating variable. Unadjusted models were fitted, as well as models adjusted for potential confounders for this association. Per capita household consumption, HSV-2 and orphan status were tested as potential effect modifiers. Overall, associations between transactional sex and each categorical potential mediating variable were assessed using the likelihood-ratio test (LRT). In this and all subsequent models, we accounted for clustering at the school level by using cluster-robust standard errors.

### Mediation analysis

We used traditional mediation analysis [45] to test whether our hypothesized variables around partnership dynamics and relationship characteristics mediated the association between transactional sex and HIV. First, we estimated the *total effect of the exposure on the outcome*, by developing a logistic regression model for the association between transactional sex and HIV, adjusting for all potential confounders. Next, we estimated the *direct effect of transactional sex on HIV* by fitting a logistic regression model that included the potential mediating variable(s) and any further exposure–mediator or mediator–outcome confounders. A comparison of the total and direct effects estimated by these two models allows an assessment of the extent to which the association is mediated by the hypothesized variable(s). Each potential mediator was first considered individually and then all mediators were considered together in the same model.

Our final models only included cases with no missing data for each of the chosen mediating variables yielding a sample of 631 sexually active participants (24.9% of total *n*=2533 or 91.1% of sexually active women, total *n*=693).

## Results

### Characteristics of the population

The age of young women in the study sample ranged from 13 to 20 years (Table 1). From the overall sample (*n*=2533), just over a quarter (*n*=693 or 27.4%) of young women reported being sexually active, of which 78.2% were between 16 and 20 years. The mean age of first sex (vaginal and/or anal sex) in this sample was 14.7 years. Close to 30% of sexually active young women reported ever being pregnant and 6.2% (*n*=43) were HIV-positive. Among sexually active young women, close to 20% lived in large households with eight or more family members and almost 40% reported that they were worried that their household did not have enough food in the past year. The primary caregiver for most young women (68.1%) was their mother and a quarter of primary caregivers had never attended school; a little over a quarter (28.3%) had completed primary school and a little less than a quarter had completed secondary school (23.7%). In terms of financial independence, 21.8% of sexually active young women reported working for cash. More than half (55.8%) of these sexually active young women cited financial independence as their main reason for working. Of the young women who were sexually active, 78.2% reported having a current boyfriend, and 45.5% of sexually active young women had at least two or more sexual partners in their lifetime and 21.2% had more than two sexual partners in the past 12 months.

Overall, 14% (*n*=97) of sexually active young women or 3.8% of young women from the entire sample (*n*=2533) reported feeling as though they had to engage in “sex for money, gifts or both” (transactional sex). The majority of transactional sexual relationships were only with the current partner (67%), in comparison with one or both previous partners. Almost 60% (*n*=57) of young women in transactional relationships reported their current partner as their main partner with the remaining 40% (*n*=40) as casual partners. The majority of items were received from primary partners with 60% having received money, 25% having received gifts (such as cosmetics or airtime) and approximately 15% having received both money and gifts.

### Unadjusted analysis between transactional sex and HIV

Of those young women who reported ever engaging in transactional sex, 12.4% (*n*=12) were HIV-positive compared with 5.2% (*n*=31) of those who did not report transactional sex. The unadjusted analysis indicates that young women who reported that they felt they had to engage in sex because they received money or gifts had increased odds of being HIV-positive (OR: 2.6, CI: 95%: 1.28–5.36, *p*=0.01) (see Table 3).

### Transactional sex and mediators (partnership characteristics and relationship dynamics)

Table 2 shows unadjusted and adjusted results from the analysis of the association between transactional sex (exposure) and each of the potential variables that are hypothesized to mediate the relationship (as indicated in Figure 3) between transactional sex and HIV. The adjusted results show that young women who reported engaging in transactional sex have three times higher odds of having sex while drunk (aOR: 3.1, CI: 95% 1.55–5.71, *p*=0.002) and almost double the odds of engaging in concurrent partnerships (aOR: 1.86, CI: 95% 1.18–2.91, *p*=0.01). They also report lower scores on the Sexual Relationship Power Scale (SRPS) relative to the high score after adjusting for confounders (aOR: 1.73, CI: 95% 0.96–3.12, *p*=0.06) compared with those who do not report engaging in transactional sex.

**Table 2:** Odds ratios from logistic/multinomial logistic regression analysis of the association between transactional sex and each mediating variable for relationship characteristics and partnership dynamics (*n*=693)

Outcome	uOR	95% CI	p*	aOR^a^	95% CI	p*
Age difference with partner^b^						
>Five years older versus up to five years older			0.37			0.51
No TS	Reference			Reference		
TS	1.35	0.69–2.66		1.33	0.40–1.58	
Same age/younger versus up to five years older			0.56			0.51
No TS	Reference			Reference		
TS	1.22	0.62–2.38		1.25	0.43–2.61	
Condom use at last sex			0.37			0.33
No TS	Reference			Reference		
TS	1.25	0.76–2.04		1.27	0.77–2.10	
Sex on alcohol or drugs			**0.01**			0.001
No TS	Reference			Reference		
TS	2.56	1.32–4.98		3.10	1.55–5.71	
Young women's partner concurrency			0.07			0.01
No TS	Reference			Reference		
TS	1.83	1.18–2.84		1.86	1.18–2.91	
Sexual Relationship Power Scale^b^						
Medium power versus high power			0.73			0.70
No TS	Reference			Reference		
TS	0.91	0.2–1.56		0.52	0.51–1.56	
Low power versus high power			0.09			0.06
No TS	Reference			Reference		
TS	1.63	0.93–2.86		1.73	0.96–3.12	
Young women's perception of partner concurrency^b^						
Concurrent partnership versus no concurrent partner			0.06			0.06
No TS	Reference			Reference		
TS	0.60	0.34–1.03		0.59	0.34–1.03	
Don't know versus no concurrent partner			0.09			0.10
No TS	Reference			Reference		
TS	0.63	0.37–1.09		0.63	0.36–1.09	
Sexual partners past 12 months						
Two partners versus one partner						
No TS	Reference			Reference		
TS	0.98	0.52–1.85	0.95	0.92	0.48–1.77	0.81
> Three partners versus one partner						
No TS	Reference			Reference		
TS	1.98	0.93–4.23	0.08	1.91	0.86–4.21	0.11

Unadjusted odds ratio estimation through logistic regression; all records with missing data excluded.

a Adjusted for confounders: per capita household consumption, educational level of primary caregiver, having a boyfriend, age of first sex, age of young women and being an orphan

b categorical variables – performed multinomial regression TS.

* *P*-value calculation through likelihood-ratio test; p<0.05 significant.

### Mediation analysis between transactional sex and HIV

Table 3 shows the unadjusted analysis (mentioned above) and the effect of transactional sex on HIV, after adjusting for potential confounders in the sub-sample of young women with no missing data on all potential mediators (total effect). After adjusting for confounders, young women who report engaging in transactional sex have two and a half times higher odds of being HIV-positive (aOR: 2.5, CI: 95% 1.19–5.25, *p*=0.01).

**Table 3:** Unadjusted analysis and effect of transactional sex on HIV adjusted for confounders among sexually active women (*n*=631^a^)

TS–HIV	uOR	95% CI	p*	aOR^b^	95% CI	p*
No	Reference		**0.01**	Reference		0.01
Yes	2.6	1.28–5.36		2.5	1.19–5.25	

uOR, unadjusted odds ratio.

a Records with missing data excluded

b adjusted for confounders (age of young woman, having a boyfriend, per capita household consumption, educational level of primary caregiver, age of first sex, orphan status and work done for money).

* *P*-value estimation through likelihood-ratio test; p<0.05 significant.

Table 4 shows the direct effect of transactional sex on HIV not mediated by the hypothesized variables, first presented by each mediator individually and then all potential mediators together, adjusted for confounders. Little difference exists between the adjusted estimates of the direct effect of transactional sex and HIV on each of the different mediators (models one to eight) with odds ratios ranging from 2.4 to 2.6. The overall adjusted model including all mediators demonstrates that young women who engage in transactional sex had almost triple the odds of being HIV-positive (aOR: 2.6, CI: 95%: 1.16–5.63, *p*=0.02).

**Table 4:** Effect of transactional sex (TS) on HIV adjusted for confounders and mediators (*n*=631^a^)

	Variables	AOR^b^	95% CI	p*
Model 1	TS**b**+Age diff with partner			0.02
	No	Reference		
	Yes	2.4	1.23–5.86	
Model 2	TS**b**+condom use at last sex			0.01
	No	Reference		
	Yes	2.5	1.27–5.93	
Model 3	TS**b**+sex under alcohol/drugs			0.01
	No	Reference		
	Yes	2.5	1.27–6.03	
Model 4	TS**b**+YW's partner concurrency			0.02
	No	Reference		
	Yes	2.4	1.22–5.77	
Model 5	TS**b**+Sexual Relationship Power Scale			0.01
	No	Reference		
	Yes	2.6	1.30–6.17	
Model 6	TS**b**+YW's perception of partner concurrency			0.01
	No	Reference		
	Yes	2.5	1.26–5.95	
Model 7	TS**b**+sexual partners last 12 months			0.01
	No	Reference		
	Yes	2.6	1.28–6.12	
Model 8	TS**b**+HSV-2 infection			
	No	Reference		
	Yes	2.5	0.98–5.35	0.02
Overall^b^	TS**b**+all mediators			0.02
	No	Reference		
	Yes	2.6	1.16–5.63	

a Records with missing data excluded

b adjusted for all confounders (age of young woman, having a boyfriend, per capita household consumption, educational level of primary caregiver, age of first sex, orphan status and work done for money)

c adjusted for all mediators (age difference with partner, condom use at last sex, sex under the influence of alcohol and drugs, young women's own partner concurrency, sexual relationship power scale (SRPS), perception of partner's concurrency, sexual partner in the last 12 months, HSV-2 infection).

* *P*-value calculated through likelihood-ratio test; p<0.05 significant.

Thus, the estimated total effect presented earlier (Table 3) of transactional sex and HIV had an odds ratio of 2.5 and the direct effect (Table 4) had an odds ratio of 2.6. Given that there is little variation in the two results, it appears that none of the hypothesized variables mediate the association between transactional sex and HIV infection.

## Discussion

This cross-sectional analysis explored the prevalence of transactional sex and the relationship between transactional sex and HIV risk among a sample of sexually active secondary school girls aged 13–20 years from rural Mpumalanga, South Africa. The results show that transactional sex was associated with almost three-fold increased odds of being HIV-positive, after controlling for other risk factors. These data are consistent with observations from other settings with young women in South Africa [10,31].

Surprisingly, however, we found that the association between transactional sex and HIV was not mediated by any of the sexual risk behaviours that might help to explain the underlying pathways of HIV risk. For example, in this study, the age difference with the partner and young women's number of sexual partners do not appear to mediate the relationship between transactional sex and HIV infection. This is counter to expectation because previous research has shown that age difference with partners is associated with higher HIV risk and that young women who engage in transactional sex tend to have more sexual partners than other women [9,46]. This lack of mediation needs to be interpreted cautiously, however, as the measures used to capture certain concepts (e.g. transactional sex or sex under the influence of alcohol/drugs) still need appropriate validation. Furthermore, given the cross-sectional nature of the data, we do not know whether any of the risky sexual behaviours are the same as they were at the time of actual infection with HIV. The findings therefore highlight the need to further explore the potential pathways through which transactional sex may increase young women's risk of HIV through longitudinal data that are collected at more than one point in time [10].

The question then arises as to what other aspects of transactional sex might make it risky for HIV. It is plausible that these relationships might be part of higher risk networks and young women are made vulnerable through the underlying risk of the men that they choose to have sex with (with high risk not being marked solely by age). This corroborates work conducted by Prudden *et al*. Their analysis suggests that young females with multiple partners serve as a network to high-risk male partners that render them vulnerable to HIV [47]. This also aligns with evidence from DHS data that suggest that paying for sex was associated with HIV-positive serostatus among young men and a higher number of lifetime sexual partners was associated with HIV-positive serostatus among young women [48]. Hence, developing an understanding of the aspects of transactional relationships that are high risk for HIV (in terms of exposure to a network of men that are considered high risk, irrespective of the age difference) needs to be explored further [41].

Our analysis also suggests that young women who reported transactional sex are more likely to have scored low on the SRPS, to ever have had sex under the influence of alcohol or drugs and to have concurrent partners. A low score on the SRPS indicates less power in terms of relationship control, negotiation or decision-making. Thus, even though the specific variables tested did not mediate the relationship between transactional sex and HIV, there may be more complex ways in which some factors (e.g. low partnership equity, lack of consistent condom use or use of alcohol) affect HIV risk. For example, the receipt of gifts or money from a partner is often a normal part of adolescent romantic relationships in sub-Saharan Africa: accessing money or items may be a key motivating factor in such unions [49,50]. It was difficult to assess whether material gain was the primary motivation for sex in the young woman's relationship because of the way the questions on transactional sex were asked in the baseline survey (“if she feels like she had to have sex to receive money and/or gifts”). Thus, depending on how the relationship is perceived by either party (as being transactional rather than gift-based) has implications for understanding power dynamics within a relationship and can explain the low score in the SRPS among young women. For example, where there is financial motivation, women may find it hard to negotiate condom use due to the material nature of the negotiation. Alternatively, when love is the primary motivation, women may either want to get pregnant or have difficulties negotiating condom use as this may suggest a lack of trust in a partner with whom they are in love [51]. In addition, research suggests that women who receive gifts or money informally have less negotiating power than sex workers who explicitly negotiate the terms of each sexual encounter. As the exchange is not openly discussed, men may feel entitled to have sex on their terms, leaving young girls and women with little power to assert their own preferences for monogamy or protected sex [10,52]. Future rounds of the HPTN 068 survey included questions around primary motivations for engaging in transactional sex in order to capture the specific risky aspects of transactional sexual relationships that are associated with HIV risk.

Furthermore, literature from Cape Town, South Africa, suggests that alcohol may affect HIV risk through means other than its direct effect on sexual inhibition [24]. Ethnographic research suggests that some young women who frequent *shebeens* (township bars) do so with the expressed intention of finding men to pay for their drinks [16] and that sexual encounters usually follow [53]. Furthermore, other cross-sectional evidence from HPTN068 suggests that frequenting alcohol outlets was associated with increased sexual risk among young women [54]. It may be that men who frequent *shebeens* have certain characteristics and behaviours that increase their risk of being HIV-positive [55]. Indeed, other studies show evidence of clustering of risks in men: those who engage in transactional relationships may be substantially more controlling, patriarchal and violent than other men [27,56]. Thus, frequenting bars may increase women's risk because it brings them in contact with these particular types of men who are more likely to be HIV-positive [11,55] and these young women might agree to riskier sex (e.g. unprotected sex), and be less able to refuse it, when drunk.

This study had a number of strengths and limitations. In contrast to other studies, this analysis is based on a biological measure of HIV, not self-reported sexual behaviours as proxy measures for HIV, which are subject to recall bias and false reports [57]. In addition, since this research was embedded in a large randomized controlled trial funded by the HIV Prevention Trials Network (HPTN), the data were subject to rigorous quality checks [33]. However, we recognize that this paper is a secondary analysis of data and that there are limitations to how certain measures, such as transactional sex, have been conceptualized and measured. Hence, we need to take this into consideration when interpreting the findings. It is also important to mention that there are currently no validated measures of transactional sex. The first and second authors are members of an international working group (www.strive.lshtm.ac.uk/themes/transactional-sex-and-hiv) to develop better measures of transactional sex, and efforts are underway to try to improve measurement using methods, such as cognitive field-based testing, but this is work in progress.

The cross-sectional nature of the data makes the assessment of causality problematic. For example, it is difficult to assess the timing of transactional sex in relation to the acquisition of HIV. In addition, as the exposure and outcome are being measured at the same point in time, it is difficult to make a definitive case for a variable being either a confounder or mediator. For example, the decision for whether number of sexual partners should be considered as a confounder or mediator depends on how the transactional sex variable is conceptualized. If transactional sex is conceptualized as something that pre-dates most sexual activity (i.e. there is some inclination to engage in transactional sex), then one can make the case for this driving the number of sexual partners, and sexual partners would be considered to be a mediator. If, however, the motivation to engage in transactional sex is driven by the number of sexual partners (i.e. the more sexual partners a young woman has, more likely she is to engage further in transactional sex), the number of sexual partners could be a confounder of the association between transactional sex and HIV. Based on the conceptual framework, we have conceptualized the number of sexual partners as a potential mediator; as intuitively given the current context in rural South Africa where economic opportunities are circumscribed, young women are inclined to engaging in transactional sex with multiple sexual partners to fulfil their wants and needs, hence putting themselves at risk for HIV.

Furthermore, the importance of social desirability bias that plays an important role in self-reported sexual behaviours might also account for the lack of mediation in our results [58]. For instance, the expected direction of social desirability bias is that respondents will over-report condom use and under-report the number of sexual partners [59]. Even the questions around transactional sex generally tends to be under-reported as, unlike female sex workers who self-identify as sex workers, young women engaging in transactional sex seldom disclose that they have exchanged sex for money. Despite the use of methods, such as ACASI in this study, which eliminate the need for respondents to report socially undesirable answers face-to-face, it is important to acknowledge the important role that social desirability bias might play when interpreting these findings and in drawing conclusions.

As transactional sex and all the potential confounding and mediating variables are self-reported, recall bias is an issue. Question time-frames were chosen to be consistent with other studies (where applicable) and to facilitate recall (*e.g. sexual partners over the past 12 months? Or condom use in the last sexual encounter?*). However, individuals seldom have perfect recall of sexual events even over short time-frames and we recognize that this is a limitation. Self-completion of the questionnaire resulted in some missing data on some items. No attempts have been made to replace missing values. However, missing data were relatively uncommon and so would not be expected to cause substantial bias in the analysis that was conducted. Misclassification of transactional sex, confounding factors and mediators could lead to bias in the estimate of the total and direct effect of transactional sex on HIV and hence to incorrect conclusions regarding the extent of mediation. However, to the extent possible, we believe that all variables used in the analysis were measured as accurately as possible given the context and so significant bias is unlikely. We are confident that the presented models have been appropriately constructed and fit the data well. However, there might still be unmeasured confounding of the transactional sex–HIV relationship that needs to be considered when interpreting findings [60].

## Conclusions

In conclusion, this paper lends quantitative support to the assertion that transactional sex is both fairly prevalent and an important factor in HIV risk among young women in South Africa. However, it calls into question the pathways put forward as mechanisms through which transactional sex increases HIV acquisition. The conceptualization and measurement of transactional sex is complex and efforts are underway to try to improve measurement in order to ensure that these measures have validity and are reliable. Furthermore, future surveys need to be supplemented with questions that capture primary motivations behind such relationships. This will enable a better understanding of aspects of transactional sex relationships that contribute to HIV risk. In addition, longitudinal studies that examine the complex pathways through which transactional sex may increase HIV risk will mitigate challenges of reverse causality from cross-sectional data. A potential explanation for what makes transactional sex risky for HIV may be the networks of sexually connected individuals who are considered “high risk” for HIV and the underlying risk of the men that young women have chosen as partners. Hence, adopting a structural approach that can alter the context of young people's HIV risk by moving beyond individual-level measures of knowledge towards addressing economic and structural factors that underlie HIV risk are important [61].

## Appendix 1

**Description and construction of variables**

**Mediating variables**

We used the following steps for deriving and categorizing each measure:

*Age difference with partner* was calculated by first asking the age of each of the three most recent sexual partners, calculating the age difference with each partner, then calculating the mean of the three age differences to obtain a single age difference variable. This was then categorized into three groups: up to five years older than young woman; more than five years older than young woman and same age or younger than young woman. For the analysis, the category “up to five years older than young woman” served as the reference group.

*Condom use at last sex* with any partner was measured as a binary variable (no/yes) from the question “Did you use a condom with [initials] the last time you had sex?”

*Sex on alcohol or drug use* was recorded as a binary variable (no/yes) and was constructed from the questions: “Have you ever had sex while you were drunk on alcohol?” and “Have you ever had sex while you were high on drugs?”

A *Sexual Relationship Power Scale* (SRPS) (12 items, Cronbach's alpha=0.83), previously shown to be associated with incident HIV among in South African women [10,15] was used to measure relationship power equity. Items included questions around relationship control and decision-making dominance. Each item was assessed on a 3-point Likert scale and the measure was scored from 0 to 24 and categorized into tertiles. For the analysis, the tertile with the lowest power equity served as the reference group.

*Young women's own partner concurrency* was recorded as binary and coded as “1” if the woman reported additional partners during any of her last three relationships. The variable was constructed from the question: “During the time that you and […] have had a sexual relationship, have you had any other sexual partners?”

*Young women's perception of her partner's concurrency* was categorical and constructed from the question for any of her three partners. “As far as you know, during the time that you and [initials] have had a sexual relationship, has [initials] had any other sexual partners, such as girlfriends, wives or sex workers?” The categories were: no (concurrent partner), yes (concurrent partner) and don't know.

*Young women's number of sexual partners in the past 12 months* was recorded from 0 to 15 and was categorized into four groups: 0, 1, 2, >3.

**Other variables**

*The age of young women* was recorded as a continuous variable from 13 to 20 years and was re-categorized into two groups of 13–15 years and 16–20 years for equal sample size in each category.

*The age of first vaginal and/or anal sex* was constructed from the questions “How old were you when you first had vaginal sex? How old were you when you first had anal sex?” and re-categorized into two groups –<15 years and 15 years and older.

Employment status of the young woman was recorded as a binary variable and constructed from the question “Did you do any work for pay or family gain, including payment in kind such as food or housing?”

*Per capita household consumption* as a measure of living standards was calculated using the module on food and non-food spending and consumption in the household questionnaire. This was done by summing all household spending and consumption on food and non-food items and by dividing it by the number of household members (total spending and consumption per capita) [44]. A categorical household consumption measure was then obtained by dividing this measure into deciles [1–10]. For this analysis, we re-categorized the variable from deciles to three groups for total amount spending/consumption per capita: low (ranging from $1.3 to $15.4), medium (ranging from $15.5 to $32.6) and high (above $32.10).

*Educational level of primary caregiver* was measured as a categorical variable with four categories: none, primary, secondary, matric (year 12) and adult basic education. We measured *orphan status* (defined as either one or both parents deceased), as binary and constructed it from the question on if the mother was alive and if the father was alive.
